# Machine learning-DeepSurv prediction model integrating mpMRI radiomics and genomic biomarkers for BCR-free survival and tumor response in prostate radiotherapy

**DOI:** 10.1093/jrr/rraf079

**Published:** 2025-12-18

**Authors:** Hossein Taheri, Mohammadbagher Tavakoli, Maryam Farghadani, Sheyda Lafzlenjani, Hamed Taheri

**Affiliations:** Hezar Jarib Street, Isfahan University of Medical Sciences, Danesh Road, School of Medicine, Ground Floor, Student Research Committe & 1st Floor, Medical Physics Department, 2nd Room, Isfahan, Iran; Hezar Jarib Street, Isfahan University of Medical Sciences, Danesh Road, School of Medicine, 1st Floor, Medical Physics Department, Head Management Room, Isfahan, Iran; Hezar Jarib Street, Isfahan University of Medical Sciences, Danesh Road, School of Medicine, Alzahra Hospital, Radiology Department, Isfahan, Iran; Hezar Jarib Street, Isfahan University of Medical Sciences, Danesh Road, School of Medicine, Alzahra Hospital, Radiology Department, Isfahan, Iran; Arak University of Medical Sciences, School of Medicine, 1st Floor, Anatomy Department, Arak, Iran

**Keywords:** prostate cancer (PCa), radiotherapy (RT), Ki-67, decipher, PTEN, radiogenomics, machine learning (ML), BCR-free survival, treatment response (TR)

## Abstract

The purpose of this study was to design a radiogenomics machine learning-DeepSurv model for biochemical recurrence-free (BCR-free) survival and treatment response (TR) prediction for radiotherapy (RT) of prostate cancer (PCa). In this study, radiomic features were extracted from pre and post treatment multiparametric MRI (mpMRI), including T_2_-weighted (T_2_W), diffusion-weighted MR imaging (DWI) and apparent diffusion coefficient (ADC). Also, genomic biomarkers such as Ki-67 (a cell proliferation marker reflecting tumor growth activity and also prognostic information in cancer progression), PTEN (tumor suppressor gene regulating cell growth and survival, have a prominent role for TR and cancer progression) and Decipher (a genomic signature predicting cancer recurrence risk and TR based on gene expression patterns) were collected. Radiomics feature selection and dimensionality reduction methods were employed, followed by training machine learning (ML) models. Moreover, time to event data and survival models including Random Survival Forest (RSF) and DeepSurv neural networks were used. For model performance, the concordance index (C-index) and integrated Brier score (IBS), and for improving interpretability, the SHapley Additive exPlanations (SHAP) were applied. Radiomic feature of MRI including Kurtosis demonstrated a near-perfect positive correlation with Ki-67 expression (r = 0.64), however skewness showed a strong negative correlation with PTEN status (r = −0.88). Entropy and kurtosis of MRI were also highly correlated with the Decipher genomic risk score (r = 0.90 and r = −0.96, respectively). The integrated ML-DeepSurve model performance overall F1-score was 0.93 for TR. The model also offered robust stratification for patients based on BCR-free survival probability. Our findings underscore the potential of radiogenomic signatures as non-invasive biomarkers to personalized PCa RT decisions and provide a novel clinically explainable predictive model based on radiomic and molecular biomarkers for BCR-free survival and TR of mentioned cancer.

## INTRODUCTION

Prostate cancer (PCa) is one of the most prevalent male malignancies around the world [1]. Image guided radiation therapy (IGRT) is a widely used treatment modality for PCa, as a result of suitable dose coverage and treatment outcome [2–5]. While, dose distribution uncertainties followed by intra- and inter-fractional variations during radiotherapy (RT), may potentially lead to differences in treatment response (TR) of the stated cancer [6–8].

Although, IGRT has emerged as a promising approach for cancer treatment, yet TR prediction of the malignancy may be unknown for each patient.

Personalized medicine opens new horizon to improve therapeutic outcomes and reduce normal tissue complications by tailoring treatment to specific patient profiles [9–11]. Furthermore, it seems that radiomics which extracts quantitative features from medical images, potentially able to characterizing tumor heterogeneity and predicting outcomes, especially when combined with pathological and clinical data [12–14]. In this regard, Zha *et al.* have demonstrated that magnetic resonance imaging (MRI)-based radiomics can effectively monitor TR in non-small cell lung cancer [15]. Besides, some studies have discussed the role of artificial intelligence (AI), including machine learning (ML) and deep learning (DL), in enhancing radiomic analysis for treatment prediction across various cancers [16–18]. In the context of PCa, radiomics has been applied to MRI for tumor grading and treatment management [19, 20]. Nevertheless, it appears that radiomics employing handcrafted typically considers merely the features of an index lesion, but PCa is a multifocal malignancy [21]. Explainability in AI refers to facilitate model decisions to be comprehensible for humans, which is critical in radiomics for developing trust and clinical adoption [22]. The Royal Society highlights that explainability is essential for radiomics models, and AI methods help to assure transparency and improve ethical use, which is vital in healthcare [22]. Shibayama *et al.* have correlated radiomic features with histopathological cell density to enable interpretable risk stratification for PCa, indicating the impact of explainable radiomics for clinical decision-making [23].

Moreover, based on some studies, genomic biomarkers have been linked to tumor aggressiveness, biochemical recurrence (BCR) and TR [18, 21, 24]. Radiogenomics, which integrates imaging and genomic biomarkers, offers a new insight toward advancing personalized treatment [18].

Many studied have shown that BCR as a strong risk factor for subsequent metastases and mortality, may predominantly develop in high-risk PCa patients (such as high initial prostate-specific antigen (PSA) levels, Gleason score (GS) ≥8, adverse RP pathology including extracapsular extension (ECE) and seminal vesicle invasion (SVI), positive surgical margins (PSM) [24].

Therefore, accurate recurrence prediction may require long-term follow-up (>5 years) after treatment due to the slow-growing and often indolent nature of PCa, and also high variability of BCR events.

Regarding to assume the TR prediction benefits for patients, we hypothesize that combining radiomic and genomic features through ML may improve TR and survival prediction for prostate IGRT. Prior studies, such as that by Leech *et al.*, have highlighted the usefulness of MRI-based radiomics for personalizing RT in PCa [25]. Also, this would seem to indicate that there is limited information about BCR-free survival AI based prediction model for PCa. Moreover, there is not enough data to draw firm conclusions about clinically explainable model for BCR- free survival using ML-DeepSurv model based on multiparametric MRI (mpMRI) radiomics features and gene expression data.

Therefore, the aim of this study is to develop ML-DeepSurv predictive model based on mpMRI radiomics and genomic biomarkers data for BCR-free Survival and TR in prostate RT.

## MATERIAL AND METHODS

### Patient selection

Eighty-five PCa patients who underwent CBCT guided RT were participated in this multicenter cross-sectional study from 2016 to 2025.

The inclusion criteria were confirmed pathologic and imaging findings and having pre- and post-treatment CT and MRI. The exclusion criteria were the lack of pre or post treatment imaging, prostate surgery and metastatic tumors. The RT dose was 2 Gy per fraction with the total prescribed dose of 45 Gy. Treatment planning was performed using Siemens, Prowess treatment planning system (TPS) for both groups.

### Imaging acquiring

Pre- and post-treatment T_2_-weighted (T_2_W), diffusion-weighted MR imaging (DWI) and apparent diffusion coefficient (ADC) (1.5 Tesla, Avento, Siemens, Germany) were acquired for all patients (TR = 3000 msec, TE = 102 msec, slice thickneess = 3 mm and inter slice gap = 1 mm).

### Radiomics feature extraction

The MRI images were used for radiomics feature extraction on the 3D-slicer software. The regions of interest (ROIs) of planning target volume (PTV) for all slices of pre- and post-RT images were also drawn. The data preprocessing including; voxel volume resampling to isotropic voxel dimensions, intensity normalization to mitigate inter-scan variability and gray-level discretization were done to streamline texture analysis. The features included first order feature, shape and texture sets were extracted employing Laplacian Gaussian (LOG) filter with sigma value of 0.5. The texture sets were the neighbor gray-tone difference matrix (NGTDM), the gray-level run length matrix (GLRLM), the gray-level co-occurrence matrix (GLCM), the gray-level size zone matrix (GLSZM) and the gray-level dependency matrix (GLDM). The least absolute shrinkage and selection operator (LASSO) modality was used for non-zero coefficients feature selection. The Least Absolute Shrinkage and Selection Operator (LASSO) method was employed for feature selection, which is a method of regulating that selects prominent features by lessening the coefficients of less relevant variables to zero. LASSO is especially effective for high-dimensional data, in which the number of features is larger than the sample size, supporting to prevent overfitting and improve generalizability of model. In this study, LASSO was employed due to its suitability for high-dimensional data and its capability to decrease overfitting. Hence, it was taken into account the noise and highly correlated features of the images were omitted and prediction accuracy was increased. The clinical characteristic features (age, prostate specific antigen (PSA), tumor stage and gleason score) and RT parameters of TPS were added to the selected features.

### Genomic features evaluation

Localized PCa is a slow-growing and often indolent nature disease. Therefore, to address this heterogeneity, genomic tools have been developed to enable tailor plans and follow-up strategies to each individual’s unique profile.

In this study the followings were evaluated:

a) Decipher™: Decipher is a genomic test that developed by GenomeDx Biosciences (Vancouver, BC, Canada) and the Mayo Clinic, which designed to estimate the risk of metastasis following PCa treatment. This genomic test was built through the analysis of 1.4 million genomic markers, including both coding and non-coding RNAs. The predictive signature of Decipher relies on 22 RNA biomarkers that are actively expressed and involved in key biological processes such as cell differentiation, proliferation, structural integrity, adhesion and motility, immune response regulation, cell-cycle control and androgen signaling.
b) Ki-67: ki-67 is a nuclear protein that linked to the synthesis of ribosomal RNA. It is commonly evaluated using semi-quantitative immunohistochemistry (IHC) as a marker for cell proliferation in cancer studies.
c) PTEN: for the mutations with well-established roles in PCa, dysregulation of PTEN is more frequently observed in advanced, localized, or metastatic cases, that has demonstrated the prognostic significance. PTEN is located on chromosome 10, and is a key component of the PI3K/AKT signaling pathway, acting as a tumor suppressor gene. Moreover, it is considered that, PTEN is the most commonly mutated tumor suppressor biomarker in PCa.

Decipher, Ki-67 and PTEN were given priority and employed in this study, due to their well-established prognostic value and validation in predicting PCa outcomes. Ki-67 is a robust proliferation marker independently associated with reduced survival and elevated likelihood of metastasis beyond conventional clinical indicators including prostate specific antigen (PSA) and Gleason score. PTEN loss is a critical tumor suppressor gene regulating cell growth and survival, have a prominent role for TR and cancer progression. In addition, the Decipher integrates a panel of biomarkers to provide a wide-ranging insight for predicting BCR and TR based on gene expression patterns. The genomic markers act in a complementary manner by underlining different aspects of tumor biology and progression, making their selection fully justified for predicting disease recurrence and guiding clinical decision-making.

### Designing machine learning model

To design prediction model, the covariate (X) was defined as selected characteristic. Furthermore, TR was considered as dependent variables (Y) using the Random Forest (RF), Decision Tree (DT), Logistic Regression (LR), Support vector machines (SVM) and K-nearest neighbors (KNN) algorithms. Internal validation was performed employing 5-fold cross-validation to avoid overfitting and model optimization. Also, external validation was conducted using an independent dataset (which was separated from the training dataset) for generalizability of the model. The independent external validation cohort was also used to evaluate the generalizability of the DeepSurv model. This external cohort consisted of patients who underwent RT at a different cancer care institute than the training cohort. To ensure compatibility, data preprocessing and feature selection methods were consistently applied across both datasets.

For cross validation, the data were divided to training and test sets. For capacity assessment of prediction model, the area under the curves (AUC) of the receiver operating characteristic curve (ROC), and also the accuracy, specificity and sensitivity were evaluated. The python software (2.7/ 3.13 [64-bit]) was applied for designing the ML model.

In addition to TR prediction, the ML-based model was developed for BCR-free survival for PCa patients post RT. In this work, time to event data including RT initiation date and BCR date or last follow-up were acquired, and survival models including Random Survival Forest (RSF) and Deep Surv neural networks were used employing the same radiogenomic data as covariates. For model performance evaluation, the concordance index (C-index) and integrated Brier score (IBS) were used. For improving interpretability, the SHapley Additive exPlanations (SHAP) were applied for identifying the key features driving individual predictions.

### Patients follow up

The included patients were followed for five years using MRI and pathology protocols based on WHO 2007.

## STATISTICAL ANALYSIS

All statistical analyses were performed using SPSS software (version 25; IBM Corp., Armonk, NY, USA) and Python scripts. Also, group comparisons were done using the independent samples t-test. Furthermore, *P*-value of less than 0.05 was considered as statistically significant. The performance of the models was assessed through receiver operating characteristic (ROC) curve analysis, including the calculation of the AUC, sensitivity and specificity. For evaluation of the relationship among genomic biomarkers and Gleason scores, Spearman’s rank correlation test was used. Finally, combined analysis of both tasks was done by calculating the diagnostic accuracy.

## RESULTS

**Figure 1:**
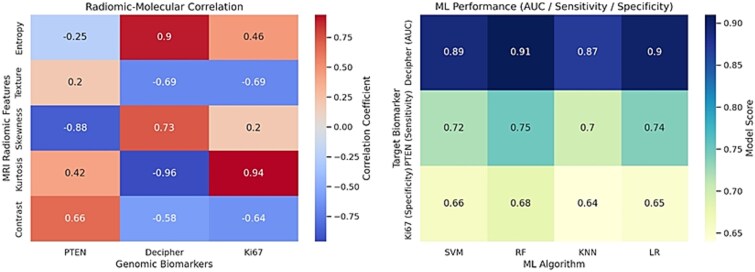
The left heatmap shows the correlation among the mpMRI radiomic features and key genomic biomarkers (PTEN, Decipher, Ki-67), highlighting the potential radiogenomic associations. The right heatmap illustrates the performance of various ML algorithms (SVM, RF, KNN, LR) in predicting the biomarkers, with the most clinically relevant metric selected for each: AUC for Decipher (overall modell discrimination), sensitivity for PTEN (detection of aggressive PTEN-loss cases), and specificity for Ki-67 (avoidance of false-positive high-proliferation prediction). This dual panel presentation supports the complementary role of radiomics and ML in the genomic biomarker prediction.

The pre IGRT characteristic features is demonstrated in Table 1. In this study also imaging extracted features including first-order statistics (18 features), shape-based (14 features) and texture features (GLCM [24 features], GLRLM [16 features], GLSZM [16 features], NGTDM [5 features] and GLDM [14 features]) were assessed (Table 2). Table 3 illustrates the correlation among radiomics features, Ki-67, PTEN expression, Decipher and Gleason Score in this study. Figure 1 shows the performance of applied ML algorithms (SVM, RF, KNN, LR) in predicting mentioned molecular biomarkers.

**Table 1:** Pre-IGRT characteristic features of patients.

	Tumor location (Transitional zone/ Parenchymal zone)	Stage	PSA	Gleason score
Intermediate grade	24/17	T1 or T2, N0, M0	10 < 20 ng/ml	7
High grade	26/18	T3 or T4, N0, M0	>20 ng/ml	≥8

**Table 2:** Extracted radiomics features

First order features	Shape based feature	Texture based feature	Texture based feature	Texture based feature	Texture based feature	Texture based feature
		Gray Level Co-occurrence Matrix (GLCM)	Gray-Level Run-length Matrix (GLRLM)	Gray-Level Size Zone Matrix (GLSZM)	Neighborhood Gray-Tone Difference Matrix (NGTDM)	Gray-Level Dependence Matrix (GLDM)
10 percentile	Elongation	Autocorrelation	Gray level non uniformity	Gray level non uniformity	Busyness	Small dependence emphasis (SDE)
90 percentile	Flatness	Cluster prominence	Gray level non uniformity normalized	Gray level non uniformity normalized		**Large dependence emphasis (LDE)**
Energy	Sphericity	Cluster shade	Gray level variance	Gray level variance	Coarseness	Gray Level non-uniformity (GLN)
Entropy	Least axis length	Cluster tendency	High Gray level non emphasis	High gray level zone emphasis	Complexity	Dependence Non-Uniformity (DN)
Interquartile Range	Major Axis length	Contrast	Long run emphasis	Large area emphasis	Contrast	Dependence non-uniformity normalized (DNN)
Kurtosis	Maximum 2D diameter column	Correlation	Long run high gray level emphasis	Large area high gray level emphasis		Gray level variance (GLV)
		-------				
		Difference average				
Maximum	Maximum 2D diameter row	Difference entropy	Long run low gray level emphasis	Large area low gray level emphasis	Strength	Dependence variance (DV)
Mean absolute deviation	Maximum 2D diameter slice	Difference variance	-	-	-	Dependence entropy (DE)
		-------				
		Id				
Mean	Maximum 3D diameter	Id_m_	-	Zone entropy (ZE)	-	Low gray level emphasis (LGLE)
Median	Mesh volume	Id_mn_	Low gray level run emphasis	Low gray level zone emphasis	-	High gray level emphasis (HGLE)
Minimum	Minor axis length	Id_n_	Run entropy	Gray level variance (GLV)	-	Small dependence low gray level emphasis (SDLGLE)
Range	Surface area	Imc1	Run length non uniformity	Size zone non uniformity	-	Small dependence high gray level emphasis (SDHGLE)
Robust mean absolute deviation	Surface volume ratio	Imc2	Run length non uniformity normalized	Size zone non uniformity	-	Large dependence low gray level emphasis (LDLGLE)
		-------	-------			
		Inverse variance	Run percentage			
		-------	-			
		Joint average	-			
Root mean squared	Voxel volume	Joint energy	Run variance	Small area emphasis	-	Large dependence high gray level emphasis (LDHGLE)
		-------	-------			
		Joint entropy	Short run emphasis			
		--------	-			
		MCC	-			
Skewness	Spherical disproportion	Maximum probability	Short run high gray level emphasis	Small area high gray level emphasis	-	-
Total energy	--------	Sum average		Small area low gray level emphasis	-	-

**Table 3:** Associations among Radiomics Features, Ki-67 (a), PTEN expression (b), Decipher (c) and Gleason Score

(a)					
Radiomics studied features	Applied ML algorithm	Correlation with Ki-67	*P*-value (Ki-67)	Correlation with gleason score	*P*-value (Gleason)
GLCM-Entropy	SVM	0.62	0.005	0.58	0.005
First-order based Mean	LR	0.48	0.011	0.43	0.04
Shape based Sphericity	RF	0.13	0.32	−0.34	0.047
NGTDM -Coarseness	Lasso	−0.39	0.045	−0.44	0.02
GLRLM RunLength	Logistic Reg.	0.21	0.19	0.26	0.15
(b)					
Radiomics studied feature	Applied ML algorithm	Correlation with PTEN	*P*-value (PTEN)	Correlation with gleason score	*P*-value (Gleason)
Skewness	RF	−0.88	0.002	−0.44	0.014
GLCM Contrast	SVM	−0.51	0.021	0.41	0.036
Kurtosis	LR	0.18	0.27	0.22	0.12
NGTDM Strength	Lasso	−0.39	0.045	−0.47	0.19
Shape based Elongation	LR	−0.25	0.14	−0.29	0.09
(c)					
Radiomics studied feature	Applied ML algorithm	Correlation with Decipher	*P*-value (Decipher)	Correlation with gleason score	*P*-value (Gleason)
GLCM Entropy	SVM	0.90	0.003	0.59	0.006
Skewness	RF	−0.46	0.018	−0.35	0.048
GLRLM ShortRunEmphasis	LR	0.29	0.09	0.22	0.13
NGTDM Coarseness	Lasso	−0.38	0.049	−0.44	0.02
Shape Elongation	LR	−0.22	0.17	−0.28	0.11

**Figure 2:**
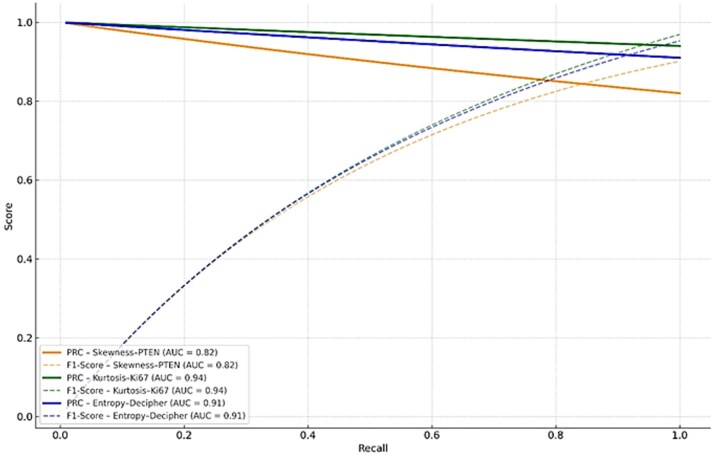
Precision-Recall and F1 score curves for radiogenomic feature-biomarker pairs of this study. Solid lines show precision-recall curves, while dashed lines correspond to F1 scores across varying recall thresholds. The Kortosis- Ki-67 pair indicated the highest predictive performance, followed by Entropy-Decipher. Skewness- PTEN demonstrated comparatively lower precision and F1 score.

Figure 2 reflects the Precision Recall and F1 score curves for radiogenomic feature–biomarker pairs in this study. Table 4 summarizes the comparative performance of mentioned ML models for the top radiogenomic pairs in terms of AUC, precision, recall, F1 score and confidence interval (CI). As demonstrated in the Tables, SVM algorithm provided more suitable biomarkers than RF, DT, LR and KNN for the MR imaging.

**Table 4:** Performance metrics of ML models for radiogenomic feature and biomarker pairs

Radiogenomic pair	ML algorithm	AUC	Precision	Recall	F1 score
Kurtosis–Ki67	SVM	0.94	0.96	0.91	0.93
Entropy–Decipher	SVM	0.91	0.89	0.88	0.88
Skewness–PTEN	RF	0.82	0.75	0.79	0.76

The table includes the AUC (Area Under Curve), precision, recall (sensitivity) and F1 score in this study. Among ML algorithms, the SVM model outperformed others for the Kurtosis–Ki67 and Entropy–Decipher pairs, however Random Forest exhibited acceptable performance for Skewness–PTEN combination.

In this study, the RSF and DeepSurv models illustrated robust performance for BCR-free survival prediction with C-indices of 0.82 and 0.85, respectively, which demonstrating the high concordance among predicted and observed outcomes. Furthermore, the Kaplan–Meier analysis (that was based on risk stratification from predicted survival probabilities) exhibited significant separation (log-rank *P* < 0.001). Moreover, the analysis of SHAP revealed that the Decipher score, PTEN status and entropy from MR radiomics were the top contributors influencing survival predictions. The SHAP analysis, for feature importance, also shown the Decipher and entropy are the top contributors.

**Figure 3:**
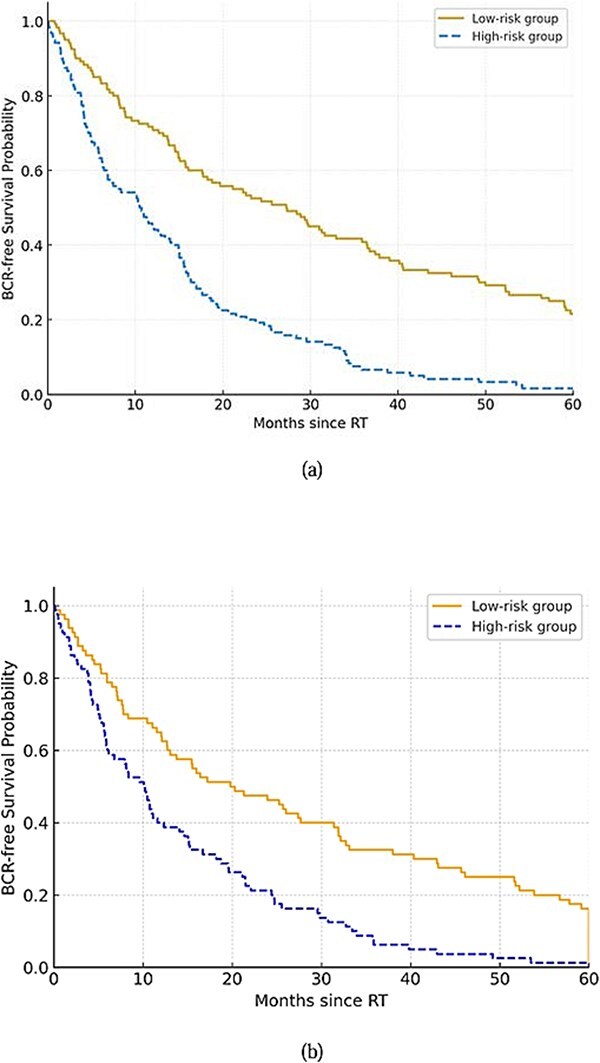
Comparison Kaplan-Meier survival curves for high-risk and low-risk prostate cancer patients in the proposed model for internal (a) and external (b) validations. The high-risk group showed significantly reduced BCR-free survival over time (log-rank < 0.001).

For evaluating the developed BCR-free survival prediction model, C-index and IBS were used. Figure 3 and Table 5 show that the DeepSurv slightly outperformed the RSF (C-index: 0.85 vs 0.82).

**Table 5:** Survival prediction performance among DeepSurv and RSF models. DeepSurv model yielded slightly higher predictive accuracy for BCR-free survival prediction compared to RSF

Applied model	Concordance index (C-index)	Integrated Brier Score (IBS)	Log-rank *P*-value
Random Survival Forest	0.82	0.18	< 0.001
DeepSurv	0.85	0.15	< 0.001

Table 6 indicates the performance comparison of studied predictors, in which the Kurtosis–Ki-67 showed the highest performance (AUC = 0.94) than others.

**Table 6:** The Kurtosis–Ki67 combination demonstrated the highest AUC (= 0.94), followed by Entropy–Decipher (AUC = 0.91) and Skewness–PTEN (AUC = 0.82). The dashed diagonal illustrates the performance of a random classifier (AUC = 0.50)

Feature	AUC
PTEN –Skewness	0.82
Ki67–Kurtosis	0.94
Decipher –Entropy	0.91
Random Classifier	0.50

**Figure 4:**
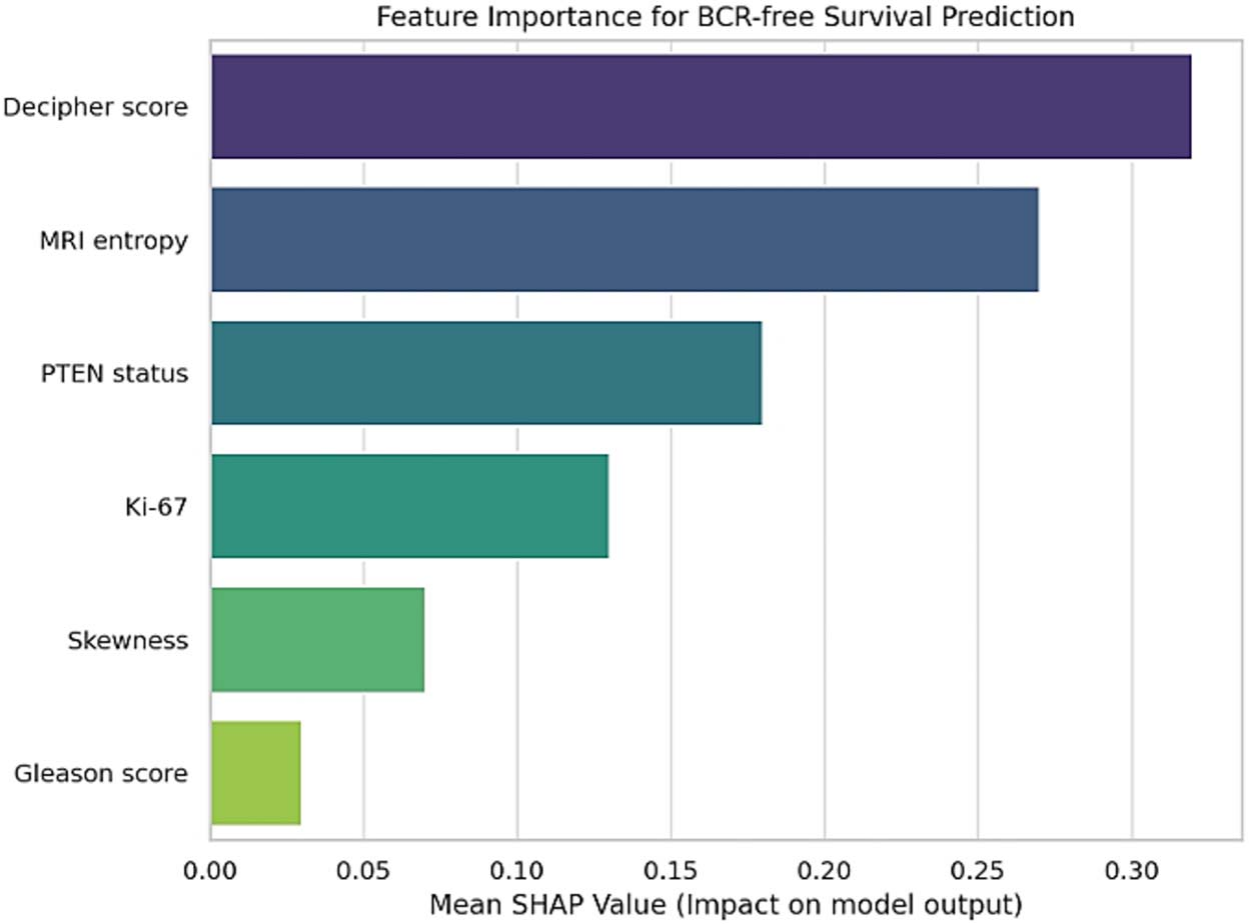
Feature importance ranking resulting from SHAP (SHapley Additive exPlainations) analysis. Decipher score and MRI entropy were the most influential features for predicting BCR-free survival among others.

Based on Fig. 4, which illustrates Kaplan–Meier curves for the developed prediction model, high- and low-risk groups, there is a statistically significant separation (log-rank *P* < 0.001). These explainable outputs also provide worthwhile foundation that supporting their potential clinical utility for clinically personalized treatment planning and follow-up strategies.

The framework indicated robust predictive performance for BCR-free survival prediction of the external validation cohort (C-index of 0.81), comparable to the performance of training datasets (C-index of 0.85). These findings confirm the generalizability of the model beyond the training sample and emphasize there is not overfitting in the model. The Kaplan–Meier curves for the external validation showed a significant separation (log-rank *P* < 0.001) (Fig. 3b).

## DISCUSSION

RT is one of the most widely employed methods for PCa, while the TR and also BCR of PCa may be varied based on patients’ specific features. Therefore, developing a dual prediction model for TR and BCR-free survival is pivotal in personalized therapeutic decisions. The radiomics, genomics and AI advancements in anticipating the BCR survival may enhance the clinical outcomes and are ushering in a new era in the tailored PCa RT.

Bodalal *et al.* have highlighted the emerging advantages of radiogenomics as a bridge between imaging phenotypes and genomic profiles, emphasizing its potential to advance personalized treatment, by integrating radiological data with molecular information to improve tumor characterization, treatment stratification and TR prediction [25]. Some studies have reported that the MRI features (increased entropy on ADC or DWI sequences) are associated with PTEN deletion [26]. Moreover, it would seem that the image based tissue heterogeneity has been associated with the expression of aggressive gene signatures captured by the Decipher genomic classifier [27–31].

In this study, we aimed to develop a radiogenomics informed ML-DeepSurv model to predict TR and BCR- free survival for PCa by analyzing mpMRI, Decipher, PTEN and Ki-67 biomarkers of the prostate.

Our findings illustrate that the radiomic features extracted from MR images may serve as non-invasive surrogates for underlying tumor genomics, including PTEN loss and Decipher genomic risk scores. Moreover, radiomics can quantify the physical and structural characteristics of tumors such as heterogeneity and shape, which may reflect underlying genetic alterations like PTEN loss or high Decipher scores. This suggests that the ML-DeepSurv radiogenomics approach has the potential to enhance risk stratification and individualized planning for PCa by linking MRI phenotypes to molecular tumor behavior.

Based on the results, the MRI radiomic features such as entropy and skewness were significantly associated with the genomic biomarkers including Decipher, PTEN and Ki-67. Notably, based on our findings, kurtosis indicated a near perfect positive correlation with Ki-67 (r = 0.63), while skewness showed a robust negative correlation with PTEN expression (r = −0.88). Furthermore, in this study, entropy and kurtosis were highly correlated with the Decipher score (r = 0.90 and r = −0.96, respectively) (Table 3). Besides, when used as inputs to ML-DeepSurv models, these features achieved strong predictive performance (Fig. 1). The simulated Precision–Recall and F1-score curves illustrates consistent and strong predictive performance across radiogenomic feature biomarker pairs. In our study, the Kurtosis–Ki-67 combination provided the highest precision at nearly all levels of recall, along with a corresponding F1 score peak, demonstrating excellent balance among sensitivity and precision. Also, entropy–Decipher indicated high and stable precision throughout, supporting its reliability as a predictor in our study. In contrast, Skewness–PTEN exhibited lower overall scores, although it remained within an acceptable predictive range. These trends support the discriminative power of radiomic MR features when aligned with relevant genomic markers (Fig. 2).

In this study, by performance comparison of the LASSO, RF, SVM and DT algorithms, it can be state that LASSO as a feature selection method efficiently reduced the data dimensionality and allowed framework to focus on most relevant features. RF exhibited superior performance (AUC = 0.85) compared to SVM (AUC = 0.79), due to its ensemble structure and better overfitting resistance. DT illustrated suitable performance with LASSO-selected features (0.76), but required precise adjustment. Thus, it appears that combining LASSO with advanced models such as RF and SVM enhanced the accuracy of predictions and model robustness.

This study introduces a novel radiogenomic based ML-DeepSurv model that integrates mpMRI radiomic features with molecular biomarkers (PTEN, Ki-67, Decipher) to biochemical survival and TR prediction for PCa RT. This approach combines these modalities to design TR and BCR-free survival prediction models that can stratify the patients before RT. Clinically, our proposed framework has the potential for patient-specific treatment planning by identifying tumors with radioresistant genomic profiles (PTEN loss or high Ki-67), enabling adaptive radiation dose escalation or alternative plans. Moreover, our findings showed that the radiomic features such as kurtosis and entropy, which strongly correlated with Ki-67 and Decipher scores, may serve as non-invasive imaging surrogates for high-risk genomic expression in PCa. These findings support the broader vision of precision RT for PCa, where imaging biomarkers can bridge the gap between genomic findings and real time treatment planning, that can be useful for personalized TR and BCR free survival prediction.

The developed clinical explainability decision-making framework facilitates the contribution of radiomic and genomic markers to predict survival outcomes. The explainability features applying SHAP allow health care professional to understand the mentioned model’s decision process, enhancing the trust and facilitating incorporation into clinical workflows that can be useful for specific patient treatment modality.

SHAP analysis which is shown in Fig. 4, revealed that the Decipher genomic score and MRI entropy were the strongest predictors for BCR-free survival, followed by PTEN status and Ki-67 expression. From a clinical point of view, these findings suggest that PCa patients with elevated MRI entropy or high Decipher scores, both reflective of unfavorable genetic predisposition and intra-tumor heterogeneity, that is possible to be at enhanced risk of BCR after prostate RT. These patients have potential to benefit from intensified treatment strategies, including escalated radiation dose, utilization of systemic therapy, or heightened monitoring during follow-up. Whereas, patients with favorable genomic patterns (such as low Decipher, preserved PTEN) and low-risk imaging phenotypes can potentially undergo de-intensified treatment strategies, thereby minimizing radiation induced toxicity. Furthermore, SHAP interpretability allows demonstration of feature contributions at the personalized patient level. This transparency level demonstrates the utility of the actionable model, bridging the gap between radiogenomics data and tailored RT planning for PCa.

Moreover, by the BCR-free survival analysis using the DeepSurv model, it was found strong distinctive capacity for internal and external validation. Our Kaplan–Meier survival curves analysis (that stratified by predicted risk groups [high-risk vs low-risk]) disclosed a significantly difference in BCR-free survival (log-rank *P* < 0.001), demonstrating the prognostic relevance of the proposed model. Therefore, these findings indicate that the proposed model can robustly distinguish patients likely to experience BCR, enabling intervention or adaptive treatment strategies. In this work, although a moderate sample size of 85 patients were included, multiple strategies were used to mitigate the risk of overfitting in the proposed ML-DeepSurv framework. Therefore, rigorous external validation on an independent dataset was utilized for the generalizability of ML models, validating that the predictive performance of the model remains stable beyond the training set. Also, five-fold cross-validation was performed during training to promise the robustness and stability of the framework, and prevent excessive fitting to particular samples. Moreover, the model complexity was meticulously controlled by selection of a limited number of inputs proportional to the sample size, including feature selection and dimensionality reduction methods, to avoid over-parameterization. Our results illustrated comparative performance metrics among the training (C-index: 0.85) and validation (C-index: 0.81), respectively, indicating stable and reproducible model behavior without evidence of overfitting.

Our findings are in line with previous studies that highlighted associations among imaging features and genomic markers in PCa. For instance, McCann *et al.* have concluded the significant correlations between Gleason score and PTEN expression employing mpMRI [32]. Likewise, Stoyanova *et al.* have demonstrated the concept of medical imaging habitats linked to gene expression profiles [29]. Some of previous studies have developed DL-based models for BCR prediction after prostatectomy, while in their study patients who underwent RT were excluded [16, 24, 33].

Hedge *et al.* have focused on pre- treatment MRI for biochemical failure prediction in high-risk PCa who were treated with combination of high-dose-rate brachytherapy and external beam RT, and it was concluded that the pre-treatment mpMRI is useful for identifying high-risk PCa males who are at higher risk of BCR following the mentioned treatment method [34].

Fernandes *et al.* have reported the potential of T2W imaging radiomics features alone to differentiate PCa patients with an increased risk of BCR, even in a clinically homogeneous cohort for recurrence prediction after RT [35]. They also mentioned that whole-prostate imaging characteristics for five years biochemical data evaluation can conceivably be employed for individualized treatment strategies.

Parker *et al.* have designed a pre-treatment prediction nomogram of biochemical control after neoadjuvant androgen deprivation and radical RT of PCa, by assessing two- and five-years PSA follow up, histological grade and clinical T stage to predict PSA-failure-free survival [36]. Although, the nomogram effectively stratifies patients by risk, it fails to adequately recognize complex patterns and multiple interactions among different variables in patients with low PSA combined with aggressive disease characteristics. Amico *et al.* have prepared a pretreatment nomogram for PSA failure-free survival after radical prostatectomy or external-beam RT for PCa employing Cox regression multivariable analysis, 1992 American Joint Committee on Cancer (AJCC) clinical stage and Gleason score [37], and it was concluded that high risk males (> 50%) for early (< or = 2 years) PSA failure could be classified based on the local treatment [37]. Even though the model offered many benefits in estimating survival, the Cox model as a semi-parametric approach, cannot properly account for complex and nonlinear dependencies among predictive factors in complex diseases such as cancer, and has limited ability to dynamically account for time-varying effects on survival.

Yang *et al.* have employed RF, SVM and KNN algorithms for prospectively predicting RT-induced rectal toxicities after two years in PCas [38]. In their work, correlated feature removal and four different feature selection techniques were used, and it was found that the RF model that enriched with radiomics and dosimetric data depicts higher performance compared to other models for toxicity prediction [38], which is in accordance with our findings.

Our study uniquely combines radiomics and genomic biomarkers with survival modeling into an explainable ML-DeepSurve clinically framework for personalized BCR-free survival prediction in prostate RT, an integration not previously evaluated in other studies. To the best of our knowledge, our study represents a novel framework integrating MRI data and molecular biomarkers for BCR-free survival and TR prediction for the mentioned cancer. This dual modeling approach that combines imaging features with molecular key markers such as PTEN expression, Decipher scores and Ki-67, enabling patient-level stratification RT by applying supervised ML models that offers clinical utility approach with potential implications for adaptive treatment and personalized strategies that open new insights towards prostate RT. Unlike some of previous studies, our approach offers a clinically applicable explainable predictive model that bridges MRI phenotypes with underlying tumor biology markers, indicating the potential of MRI heterogeneity metrics in predicting gene expression-based risk classifiers, that can be applied for individualized TR and BCR-free survival prediction.

One of the limitations of this study was the inability to follow-up patients beyond five years, as a result of restricted access to comprehensive extended clinical data. Follow-up time of five years is widely accepted in cancer evaluation studies as a standard benchmark, because it reflects a crucial timing window in which most BCR and disease progressions typically exhibit. This period of time allows to consider a sufficient amount of relapse related events to survival analysis with reasonable statistical power and enhances the effectiveness of treatment modality. Furthermore, it provides relevant and timely insights for therapeutic decision-making, as many treatment-related complications and functional outcomes emerge within this timeframe. However, low to intermediate-risk forms of the cancer is characterized by a relatively indolent clinical course, and late adverse events may occur well beyond five years after completion of RT. While longer follow-up periods can offer deeper insights into late cancer recurrence and survival outcomes, the robust and precisely characterized five-year follow-up data presented in our work provide valuable clinical findings on progression and prognosis of the disease.

Therefore, studies with extended follow-up periods are recommended to evaluate the durability of RT effects, late toxicities and recurrences.

## CONCLUSION

In conclusion, this study demonstrates developing a non-invasive TR& BCR-free survival prediction model based on ML-DeepSurv radiogenomics approach using mpMRI radiomic features and key genomic biomarkers including PTEN, Decipher and Ki-67 for PCa RT. Based on the results of this study, the strong correlations between radiomic-genomic biomarkers along with the robust predictive performance of the stated model, highlight the clinical utility of our findings.

In this study, the integration of explainable BCR-free survival predictions into clinical decision support systems can heighten personalized management of prostate RT. Identifying PCa patients with elevated risk of recurrence can tailor treatment methods to guide individualized RT strategies. This proposed prediction model may improve treatment outcomes and the RT management of this cancer, paving the way toward more precise and personalized PCa care.
